# Loss of Extended Synaptotagmins ESyt2 and ESyt3 does not affect mouse development or viability, but in vitro cell migration and survival under stress are affected

**DOI:** 10.4161/15384101.2014.943573

**Published:** 2014-10-30

**Authors:** Chelsea Herdman, Michel G Tremblay, Prakash K Mishra, Tom Moss

**Affiliations:** 1 Laboratory of Growth and Development; St-Patrick Research Group in Basic Oncology; Cancer Division of the Quebec University Hospital Research Centre; Québec, QC, Canada;; 2 Department of Molecular Biology; Medical Biochemistry and Pathology; Faculty of Medicine; Laval University; Québec, Canada

**Keywords:** Extended-Synaptotagmin, Esyt1, Esyt2, Esyt3, expression analysis, genetic deletion, phenotypic analysis, cell migration defects, cell survival defects, signal transduction

## Abstract

The Extended Synaptotagmins (Esyts) are a family of multi-C2 domain membrane proteins with orthologs in organisms from yeast to human. Three Esyt genes exist in mouse and human and these have most recently been implicated in the formation of junctions between endoplasmic reticulum and plasma membrane, as well as the Ca^2+^ dependent replenishment of membrane phospholipids. The data are consistent with a function in extracellular signal transduction and cell adhesion, and indeed Esyt2 was previously implicated in both these functions in Xenopus. Despite this, little is known of the function of the Esyts in vivo. We have generated mouse lines carrying homozygous deletions in one or both of the genes encoding the highly homologous Esyt2 and Esyt3 proteins. Surprisingly, *esyt2^−/−^/esyt3^−/−^* mice develop normally and are both viable and fertile. In contrast, *esyt2^−/−^/esyt3^−/−^* mouse embryonic fibroblasts display a reduced ability to migrate in standard in vitro assays, and are less resistant to stringent culture conditions and to oxidative stress than equivalent wild type fibroblasts.

## Introduction

**Figure 1.**
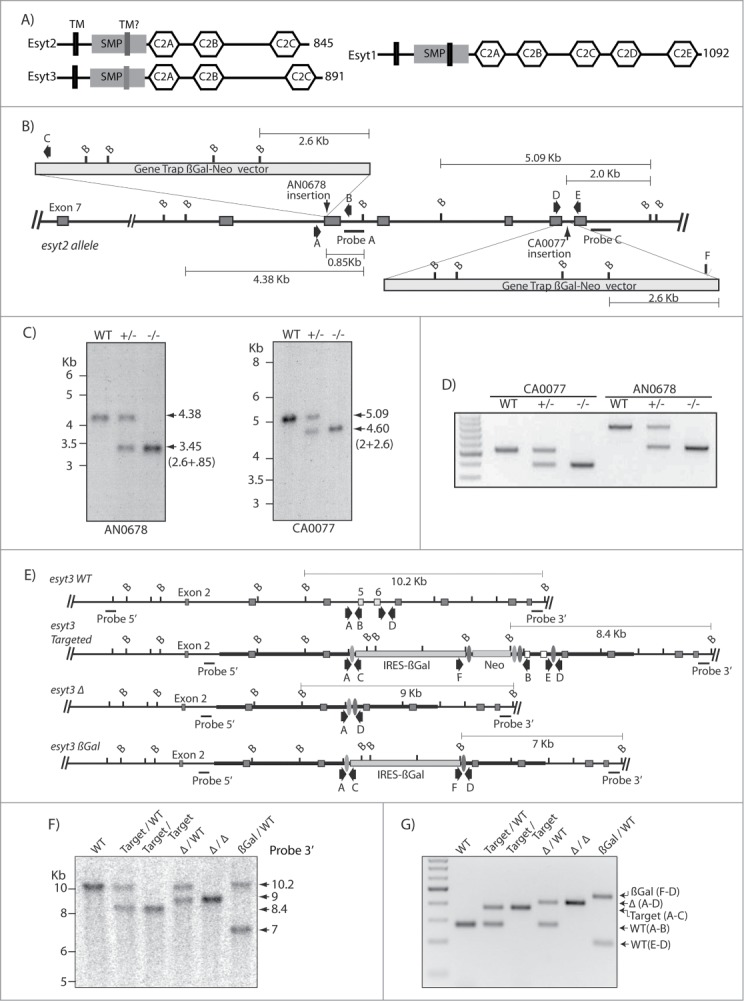
For figure legend, see page 2620.

The Extended Synaptotagmins (Esyts) are multiple C2 domain containing membrane proteins. The first member of this family of proteins was isolated from preparations of plasma membranes and high density microsome fractions of rat adipocytes.^1^ However, the Esyts were not further considered until 2007, when the primary structures of the 3 human Esyts1 to 3 were determined and their membrane associations investigated.^2^ Human Esyt1 was shown to contain 5 C2 domain homologies, while human Esyts 2 and 3 each contain 3. The C2 domains are preceded by a ∼300a.a. N-terminal region containing one or 2 putative membrane spanning domains and a predicted SMP domain ^3,4,5^ (**Fig. 1A**). Solution studies of the C2 domains of Esyt2 have confirmed their structural identity and shown that when linked they exhibit calcium dependent multimerization, while the domains display different abilities to coordinate Ca^2+^.^6,7,8^ The C2C domains of Esyt2 and 3 interact with phospholipids driving the recruitment of these Esyts to phospholipids within the plasma membrane.^2,9^ In previous studies, we identified Xenopus ESyt2 as an endocytic adapter that determines the timing of ERK activation in blastula embryos by binding both Fibroblast Growth Factor Receptor (FGFR) and Adaptin 2 (AP-2) to catalyze rapid receptor endocytosis via the Clathrin pathway.^9^ We further showed that ESyt2 recruits the cytoskeleton regulator p21-Activated-Kinase-1 (PAK1) to modulate local actin polymerization,**^10^** a function required during endocytosis.^11^ Most recently it was shown that the ESyts and the related yeast Tricalbins are in fact found inserted into the endoplasmic reticulum (ER) at sites of contact with the Plasma Membrane (ER-PM junctions).^12,13,14^ This has given rise to the model of the ESyts as 2-pass ER membrane proteins that link the ER to the PM via a C2C-domain-PtdIns(4,5)P_2_ interaction. Most recently, ESyt1 was shown to stimulate the formation of ER-PM junctions in a Ca^2+^-dependent manner, and in this way to promote recruitment of the phosphatidylinositol transfer protein (PITP) Nir2 and phospholipid incorporation into the PM.^14^

To date the demonstration that Esyt2 is required for mesoderm formation in early Xenopus embryos remains the only demonstrated biological requirement for any of the Esyt proteins. The mouse and human genomes encode 3 Esyt proteins, one of which represents the obvious ortholog of Xenopus Esyt2. In order to relate our studies in Xenopus to the apparently more complex mouse and human situation we have studied the requirements foResyt2 and 3 in mouse and in cultured mouse cells. Unexpectedly, we find that inactivation of either or both the genes for the highly similaResyts -2 and -3 has no discernible effect on mouse development, viability, reproduction or longevity. However, *esyt2^−/−^* and *esyt2^−/−^/esyt3^−/−^* embryonic fibroblasts (MEFs) display defects in both migration and resistance to culture stresses consistent with the previously proposed functions in growth factor response and the cytoskeleton regulation.

## Results

### Targeted disruption of the mouse ESyt2 and ESyt3 genes

ES cells carrying insertions in the *esyt2* gene (#CA0077 and AN0678, International Gene Trap Consortium (IGTC)) (**Fig. 1B–D**), and “Knockout First” ES cells carrying a potentially conditional insertion in *esyt3* (EPD0458_5_A10, European Conditional Mouse Mutagenesis Program (EUCOMM)) (**Fig. 1E–G**) were used to generate chimeric mice. Southern blotting and targeted PCR analysis showed that transmission of the mutant alleles was obtained in each case.

### ESyt2, −3 and 2/3 null mice are viable

**Figure 2.**
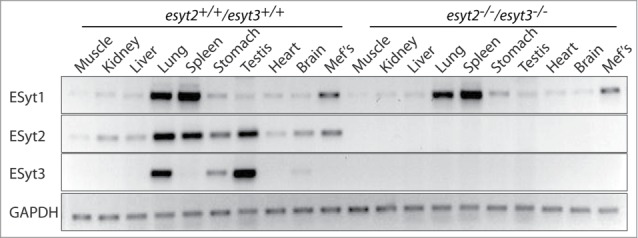
Expression of Esyt1, −2 and −3 mRNA in adult mouse tissues and MEFs. RT-PCR analyzes are shown for tissues from both wild type *esyt2^+/+^/esyt3^+/+^* and *esyt2^−/−^esyt3^−/−^* mice as compared with GAPDH.

We found that not only were the *esyt2*^−/−^ and *esyt3*^−/−^ mice viable, but the frequency of wild-type, heterozygous and homozygous null genotypes followed a Mendelian pattern of inheritance (**Table 1A**). Moreover, we found that the *esyt2*^−/−^ and *esyt3*^−/−^ mice were fertile, produced litters of normal size and did not show any overt morphological defects compared to their heterozygous and wild-type littermates. When *esyt2*^−/−^ and *esyt3*^−/−^ mice were crossed they also generated viable *esyt2*^−/−^/*esyt3*^−/−^ offspring at near Mendelian ratios (**Table 1B**). The ratios did however show some skewing toward *esyt2*^+/−^/*esyt3*^+/−^ double heterozygotes at the expense of *esyt2*^+/+^/*esyt3*^+/−^ and *esyt2*^−/−^/*esyt3*^−/−^, suggesting minor effects on viability during development. As expected, the *esyt2*^−/−^/*esyt3*^−/−^ mice expressed no detectable level of the corresponding mRNAs, but continued to express ESyt1 mRNA at wildtype levels (**Fig. 2**). *Esyt2*^−/−^, *esyt3*^−/−^, and *esyt2*^−/−^/*esyt3*^−/−^ mice also displayed a normal life span, several being kept for 18 months with no premature signs of senescence. Thus, the *esyt2* and *esyt3* genes are not essential for mouse development, viability, survival or reproduction.

**Table 1.** Genotype analysis of the progeny born A) from *esyt2*^+/−^/*esyt2*^+/−^ (alleles CA0077 and AN0678) and *esyt3*^+/−^/*esyt3*^+/−^ crosses, and B) from *esyt2*^+/−^/*esyt3*^+/−^ crosses as compared with the expected Mendelian frequencies

A)									
**Gene**	**Number of pups**	**WT/WT**	+/−	−/−					
ESyt2 (CA0077)	171	41 (24%)	86 (50%)	44 (26%)					
ESyt2 (AN0678)	180	53 (29%)	87 (48%)	40 (22%)					
ESyt3	143	18 (23%)	43 (55%)	17 (22%)					
	% Expected	25%	50%	25%					
**B) ESyt2/ESyt3**									
Number of pups	+/+ +/+	+/+ +/−	+/+ −/−	+/− +/+	+/− +/−	+/− −/−	−/− +/+	−/− +/−	−/− −/−
143	10 (7.0%)	17(11.9%)	9(6.3%)	10(7.0%)	45(31.5%)	15(10.5%)	12(8.4%)	18(12.6%)	7(4.9%)
% Expected	6.25%	12.50%	6.25%	12.50%	25.00%	12.50%	6.25%	12.50%	6.25%

### Expression pattern of the ESyts in mouse adult tissues

Expression of the *esyt2* and particularly of *esyt3* genes were found to be highly tissue specific in adults. ESyt2 mRNA was predominantly detected in lung, spleen, testis and stomach, and at much lower levels in all the other tissues tested (**Fig. 2**). The same tissue specific expression pattern was reflected foResyt1 mRNA with the sole exception of testis, which showed low levels of ESyt1 mRNA. In contrast, E-Syt3 mRNA was only expressed strongly in lung and testis, and was present at low levels only in stomach and possibly brain. The strongly overlapping expression profiles may provide some explanation for the lack of an ESyt2/3-null phenotype if ESyt1 can functionally replace the other 2 ESyts.

### Expression of ESyt2 and 3 in mouse embryos

**Figure 3.**
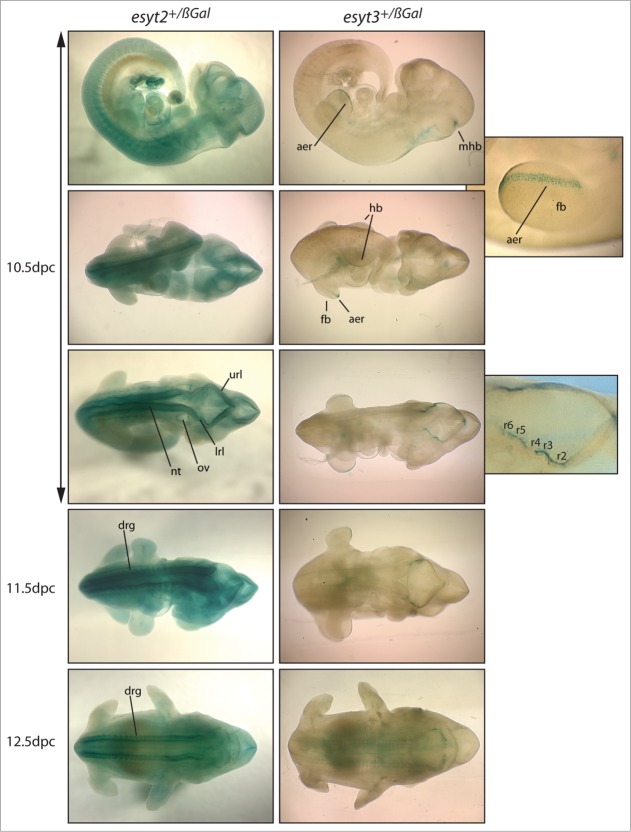
Expression pattern of the *esyt2* and −*3* genes in early mouse embryos. Expression was determined by conversion of X-Gal (blue-green) by ß-galactosidase produced from the gene inserted into the *esyt2* and *esyt3* gene loci. Enlarged panels on the right show a limb-bud and the hindbrain region of *esyt3^+/ß−Gal^* embryos. “aer” apical ectodermal ridge, “mhb” midbrain-hindbrain boundary, “fb” forelimb bud, “hb” hindlimb bud, “url” and “lrl” upper and lower rhombomere lips, “r2-6” rhombomeres, “ov” otic vesicle, “nt” neural tube, “drg” dorsal root ganglion.

It was possible that the lack of a developmental phenotype simply correlated with a lack of expression of *esyt2* and/or *−3*. However, using hetero- and homozygous embryos expressing β-galactosidase from the respective endogenous gene promoters we found that the *esyt2* gene was expressed throughout the 10.5 to 12.5 dpc embryo with little regional specificity. Expression was, however, highest in the neural tube and later in the dorsal root ganglia (**Fig. 3**). In complete contrast, at 10.5 dpc the *esyt3* gene was expressed only at the midbrain-hindbrain border (mhb), at the level of the rhombomeres (r2-r6) possibly within the cranial ganglia, and at the apical ectodermal ridge (aer) of the forelimb bud (fb) and probably the hindlimb bud. The apical ectodermal ridge is a well-documented site of FGF signaling, FGF from this region is required to maintain cell proliferation in the underlying mesenchyme.^19^ The specific expression of ESyt3 in this region, therefore, provides a tentative link with the demonstrated function of ESyt-family members in FGF signaling during early Xenopus development.^9^ The broad and strong embryonic expression of ESyt2 could explain why ESyt3 inactivation causes no obvious phenotype, but similar studies of ESyt1 will be necessary to determine if its embryonic expression is sufficiently broad to compensate for loss of both ESyt2 and 3.

### Esyt2 and Esyt3 deficiency does not impair organ development

**Figure 4.**
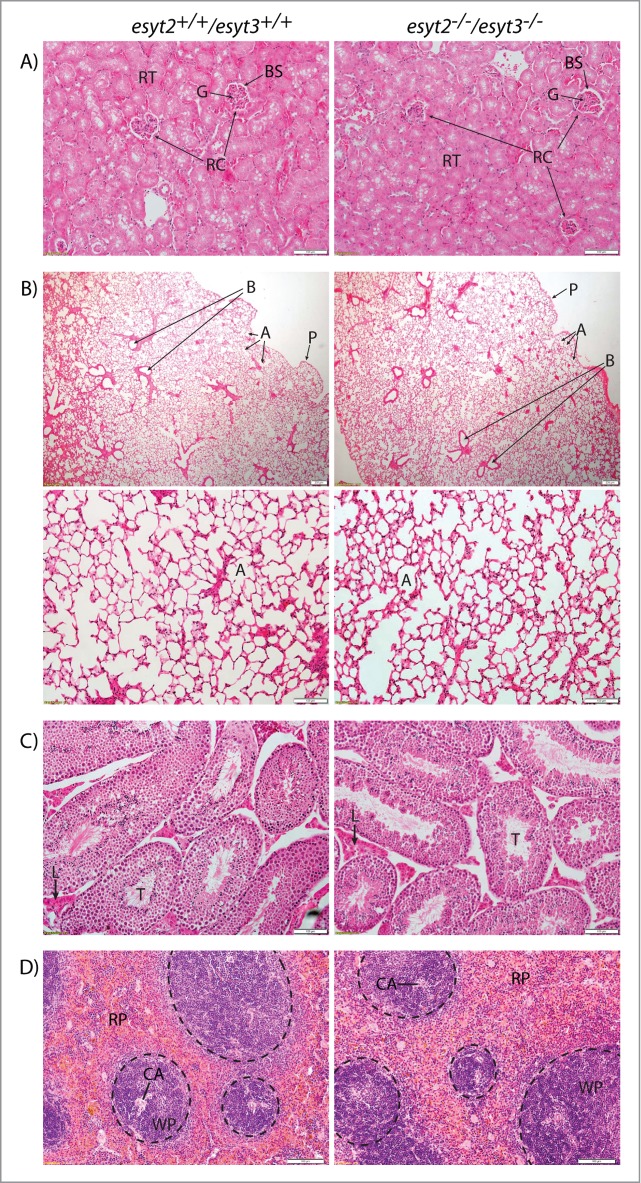
Representative Hematoxylin-Eosin staining of sections obtained from 2 11 month old *esyt2^−/−^esyt3^−/−^* sibling males and 2 11 month old *esyt2^+/+^esyt3^+/+^* sibling males. Organs displayed were those that showed a strong or differential expression of ESyt1, 2 and 3, see Figure 2. (**A**) The renal cortex of WT and DKO mice are essentially indistinguishable. Renal tubules (RT) and renal corpuscles (RC) with glomeruli (G) and Bowman's space (BS) are indicated. Scale bars 100 μm. (**B**) Top panels: Lung sections with pleura (P), bronchioles (B) and alveoli (A) indicated, scale bars 200 μm. Bottom: higher magnification of alveoli, scale bars 100 μm. (**C**) Testis morphology also appears normal, seminiferous tubules (T) and the surrounding Leydig cells (L) are indicated, scale bars 100 μm. (**D**) White pulp (WP- encircled) with central arteries (CA) and red pulp (RP) of spleen samples are indicated, scale bars 100 μm.

It was possible that the ESyt2/3 null mice harboured minor organ defects that did not affect their viability. Hence, we studied the structure of a range of organs from adult mice. However, we failed to detect anything unusual in the histology of lung, testis or spleen, in which ESyt2 and 3 are strongly expressed, or kidney, in which ESyt1 and 2 are expressed only weakly and ESyt3 was not detected (**Fig. 4**). Similarly, cursory inspection of brain and muscle histology detected no abnormalities (data not shown).

### ESyt2 loss does not affect FGF activation of ERK in MEFs

**Figure 5.**
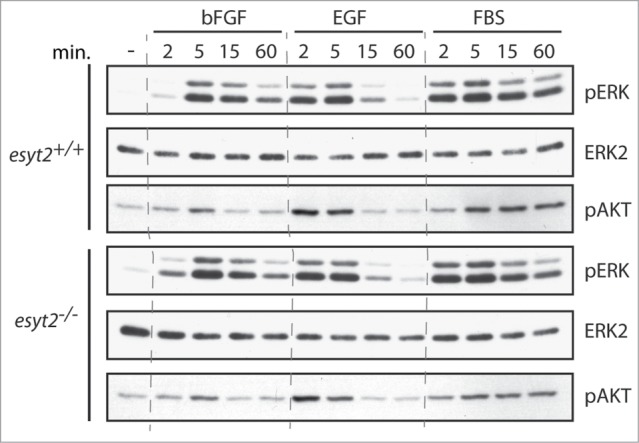
Response of ERK and AKT to extracellular stimulation in *esyt2^−/−^* MEFs. *Esyt2^+/+^* and *esyt2^−/−^* MEFs were treated at 0 min. with FGF, EGF and FBS and activation of ERK and AKT followed at the indicated times using phospho-specific antibodies (pERK (−1 and −2) and pAKT). ERK2 was detected using a specific antibody and was used as loading control.

Given that previous data had implicated Xenopus ESyt2 in FGF signaling in early Xenopus embryos,^9^ we generated embryonic fibroblasts from both ESyt2 and ESyt2/3 null mice and studied their response to FGF and other stimulations. As shown in **Fig. 2**, MEFs do not contain ESyt3 mRNA, hence we first determined whether or not activation of signaling pathways were affected in *esyt2^−/−^* MEFs. After overnight serum withdrawal, FGF, EGF and serum (FBS) induced robust and similar levels of activation of ERK and AKT in both *wt* and *esyt2^−/−^* MEFs (**Fig. 5**).

### ESyt2/3 loss does affect migration of MEFs and their viability under stress

**Figure 6.**
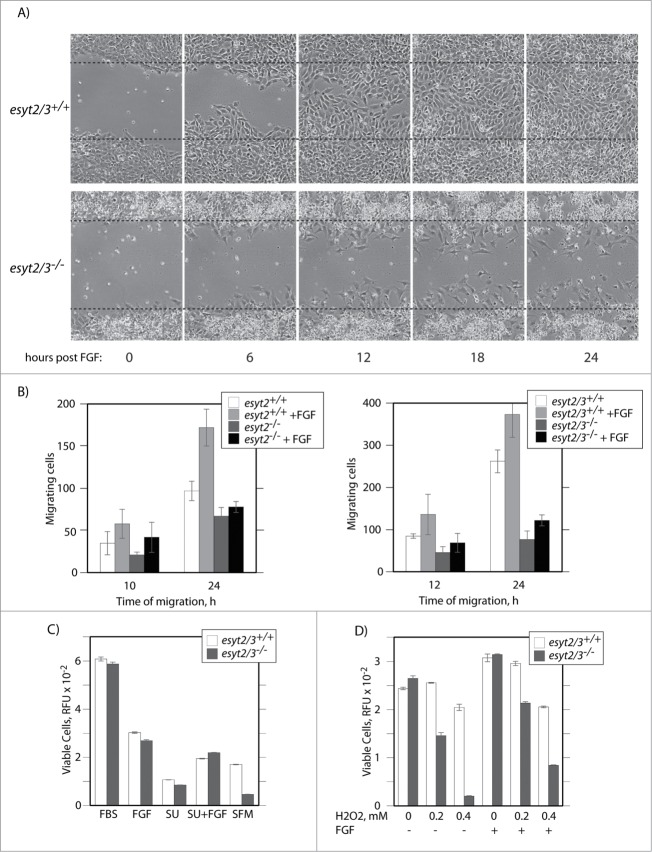
For figure legend, see page 2626.

Despite the lack of effect on signal transduction, “scratch-test” assays to determine the ability of cells to migrate when stimulated by FGF revealed that both *esyt2^−/−^esyt3^−/−^* MEFs (**Fig. 6A**) and the *esyt2^−/−^* MEFs (not shown) tended to migrate in a far less coordinated fashion and maintained little cell-cell contact during their migration as compared to *wt* (*esyt^+/+^esyt3^+/+^*) MEFs. The *esyt2^−/−^* and *esyt2^−/−^esyt3^−/−^* MEFs also migrated far less rapidly (**Fig. 6A & B**). As would be expected given the lack of esyt3 expression in MEFs (**Fig. 2**), *esyt2^−/−^esyt3^−/−^* MEFs displayed the same migration defect as the *esyt2^−/−^* MEFs (**Fig. 6B**).

The *esyt2^−/−^esyt3^−/−^* MEFs were also significantly less resistant to serum withdrawal or oxidative stress as compared to the *wt* ones. Withdrawal of serum over 4 d of incubation caused a 75% reduction in viability in *wt* MEFs, while less than 3% of *esyt2^−/−^esyt3^−/−^* MEFs survived this treatment (**Fig. 6C**). Despite this, FGF afforded a similar level of protection in both cell types, consistent with its ability to activate signaling pathways in both. The *esyt2^−/−^esyt3^−/−^* MEFs were also extremely sensitive to oxidative damage as compared to the *wt*, and again here FGF provided some degree of protection in both cases. These data show that inactivation of the *esyt2^−/−^* and *esyt2^−/−^esyt3^−/−^* genes does indeed affect aspects of cell migration and viability. These defects must, however, be compensated for in the in vivo context of the mouse.

## Discussion

Given the apparent importance of Esyt2 during Xenopus development and the recent demonstrations of the role of the Esyts in ER-PM junction formation and phospholipid generation, the lack of phenotypic effects due to the loss of Esyt2 and 3 in mouse was fully unexpected. It is, however, not without precedent. Yeast contains 3 Tricalbin (Tcb) proteins that are structurally closely related to the mammalian Esyts.^13^ Deletion studies in yeast of the Tricalbin family show that they are highly functionally redundant and in concert with other membrane tethering proteins they promote ER-PM junction formation.^13,20,21^ Indeed, deletion of all 3 Tcbs was not in itself sufficient to eliminate ER-PM tethering and this required deletion of 3 other proteins, Ist2 (a TMEM16 ion channel family member) and the vesicle-associated membrane protein-associated protein (VAP) orthologs Scs2 and Scs22. We previously demonstrated a requirement for Xenopus Esyt2 in FGF signal transduction, receptor endocytosis and mesoderm induction.^9,10^ Why very early Xenopus development was sensitive to Esyt2 depletion, while mouse is clearly not, is still unclear. This said, the expression profiles of the Xenopus Esyts suggest that only Esyt2 mRNA is present maternally (NCBI Unigene EST_Profiler, Xenbase).^22^ Thus, Esyt2 may be the only family member present during early cleavage divisions.

Despite the apparent lack of a requirement foResyt2 and -3 in mouse, MEFs carrying homozygous deletion of one or both genes display clear migration deficits in scratch test・assays and are significantly more susceptible to stringent culture conditions and to oxidative stress than otherwise isogenic *wt* MEFs. Given the connection with the PAK1 function, it is tempting to suggest that this is due to defects in cytoskeletal dynamics. We note that mRNA levels of Esyt1, the only remaining Esyt in the *esyt2^−/−^* and *esyt2^−/−^esyt3^−/−^* MEFs, are low. Possibly then this level of Esyt1 is insufficient to compensate. Clearly, these issues will only be resolved by the generation of Esyt1-null and possibly Esyt1/2/3 null mice.

## Materials and Methods

### Genotype analysis of targeted ES cells and mice

*esyt2*^+/^*^−^* (gene trapped clones Esyt2^Gt(AN0678)Wtsi^ and Esyt2^Gt(CA0077)Wtsi^) and *esyt3^+/−^* (targeted clone Esyt3^tm1a(EUCOMM)Wtsi^) embryonic stem (ES) cells were generated respectively by SIGTR and EUCOMM from Wellcome Trust Sanger Institute with the targeting vectors shown in **Fig. 1**. These clones were each used to generate 2 independent mouse lines. Southern blot analysis was used to determine the genotype of single *esyt2* or *esyt3* mutant ES cell lines and mice. For the Esyt2 clone AN0678, genomic DNA was restricted with BamHI and probed with a ^32^P-labeled 400bp probe isolated from a region located between exons 9 and 10. For the Esyt2 clone CA0077, genomic DNA was restricted with BamHI and probed with a ^32^P-labeled 510bp probe isolated from a region located immediately after exon 13. For the *ESyt3* locus, BamHI-restricted genomic DNA was analyzed using a ^32^P-labeled 380 bp 3′ probe subcloned from a region immediately after exon 10 (**Fig. 1**), and EcoRV restricted DNA was analyzed using a 350bp 5′ probe from intron 2 (data not shown). Subsequently, genotypes of animals born from crosses of *esyt2^−/−^* and *esyt3^−/−^* mice were determined by PCR amplification of genomic DNA. For clone AN0678, primers AN-A (5′-CCAATCAGCAGTCTTACCAT), AN-B (5′-CGTCTCAAGGGAAGGAAATAA) and AN-C (5′-CGCCATACAGTCCTCTTCAC) were used. Primers AN-A and AN-B amplified a fragment of 803 bp from the wild type allele whereas primers AN-A and AN-C amplified a fragment of 541 bp from the targeted allele. Primers CA-D (5′-GTTCACTCTGGACGAGGTT), CA-E (5′-CAGCTCTGATGTCTGCCAGCA) and CA-F (5′-GTAAGGAGAAAATACCGCATC) were used for the CA0077 clone. A 513 bp fragment from the wild type allele was amplified with oligonucleotides CA-D and CD-E whereas primers CA-E and CA-F amplified a fragment of 383 bp from the targeted allele. For *esyt3*, primers A (5′- CTGAAGCCTCCCAGTAGGTG), B (5′-CCATCACCCCTAGTTGTTGC), C (5′-CCACAACGGGTTCTTCTGTT), D (5′-GAGGCTCCAGGCCTTAGTTT), E (5′-CAAAAGGCAACCTCAAGGAG) and F (5′- CGGTCGCTACCATTACCAGT) were used. Primers A and B amplified a fragment of 275 bp from the wild type allele, primers A and C amplified a fragment of 367 bp from the targeted allele, primers A and D amplified a fragment of 400 bp from the delta (Δ) allele, the primers E and D amplified a fragment of 200 bp and 180 bp from the wild type and the targeted allele and the primers F and D amplified a fragment of 446 bp from the β-gal allele. The mice were housed and manipulated according to the guidelines of the Canadian Council on Animal Care and experiments were approved by the institutional animal care committee.

### Gene expression analysis by RT-PCR

Total RNA was extracted from mouse tissues using Trizol (Invitrogen) and quantified by absorbance at 260 nm. Two μg of total RNA was reverse transcribed using random primers (GE Healthcare) and mMLV reverse transcriptase (Invitrogen). PCR was performed using the primers designed with Primer3 (Untergasser et al. 2007) and the number of PCR cycles was optimized to be within the linear range of amplification. The primers used were: mESyt1.FOR (5′-TGGGATCCTGGTATCTCAGC), mESyt1.REV (5′-CTGGGAGATCACGTCCATTT), mESyt2.FOR (5′- CGAATCACCGTTCCTCTTGT), mESyt2.REV (5′- GCTCTGGAAGATTTGGTTGC), mESyt3.FOR (5′- CAAGCCCTTCATAGGAGCTG), mESyt3.REV (5′- AGCAAATGGACTCGGATCAC), mGAPDH.FOR (5′- AACTTTGGCATTGTGGAAGG), mGAPDH.REV (5′ ACACATTGGGGGTAGGAACA). Amplicons were of the expected sizes of 296 bp foResyt1, 192 bp foResyt2, 246 bp foResyt3 and 223 bp for GAPDH. Products were sub-cloned and sequenced to confirm their specificity.

### X-gal staining

Mouse embryos were isolated at E10.5 to E12.5 and fixed for 30 minutes in 1% Formaldehyde, 0.2% Gluteraldehyde, 0.02% NP-40 in 1 x PBS, washed 3 times 20 min. each in Wash Solution (2 mM MgCl2, 0.02% NP40, 1 × PBS). Embryos were protected from light and incubated overnight at R/T in the Staining buffer solution (5 mM potassium ferricyanide, 5 mM potassium ferrocyanide and 1 mg/ml X-gal in Wash Solution). Embryos were rinsed 3 times, 20 min. each, in 1 x PBS. Clarification was performed with “Sca*l*e” solution as described previously.^15^

### Histopathology

Organs were dissected from 11-month old adult mice and fixed for more than 24 hours in 4% paraformaldehyde in PBS. Samples were progressively dehydrated and embedded in paraffin. Cross sections of 5 to 20 microns were cut and stained with hematoxylin and eosin.

### Cell culture and migration assay

Primary mouse embryo fibroblasts (MEFs) from E14.5 embryos were prepared as described^16,17^ and cultured in Dulbecco's modified Eagle medium (DMEM) – high glucose (Invitrogen), supplemented with 10% fetal bovine serum (FBS, Wisent) and Penicillin/Streptomycin/Antimycotic (Anti-Anti, Invitrogen). The effects of ESyt2 and ESyt3 loss on MEF's migration were determined in a wound-healing assay (Scratch Test).^18^ Cells were seeded in a multi-6 well plate, 12 h later serum was withdrawn and cells incubated for a further 16 h before the assay. After scratching with a 2μl pipette tip, cells were incubated for the indicated times in the presence or absence of 20 ng/ml bFGF (Sigma-Aldrich) and 5 μg/ml heparin (Sigma-Aldrich). Images were taken using a Nikon TE2000 inverted microscope.

Cell viability was also determined after serum withdrawal, inhibition of FGF signaling and oxidative stress. On day 0, cells were seeded at a density of 75,000/well in 6-well plates. On day 1, cells were rinsed twice with serum-free and antibiotic-free medium (SFM) and then cultured for 6 h in the same medium. Culture medium was replaced with SFM alone or supplemented by 10% FBS, bFGF (5 μg/ml heparin, 20 ng/ml bFGF (Invitrogen)), 25 μM SU5402 (EMD/Merck), or bFGF plus SU5402. On day 3 cells were briefly rinsed twice in SFM and cultured until day 5 in fresh aliquots of the respective media. Finally, media were replaced with PBS containing 0.001% resazurin (Sigma-Aldrich) and incubated for a further 2 h before estimating the viable cell count using the relative fluorescence units (RFU) of resofurin in the cell supernatant (ex. 544 nm, em. 590 nm, Fluoroskan Ascent, Thermo Biolabs). The effects of oxidative stress were measured in a similar way, except that on day 1, cells were treated for 2 h with the indicated concentrations of H_2_O_2_ or H_2_O_2_ plus bFGF in SFM. Cells were then briefly rinsed twice in SFM before addition of medium containing 10% FBS. At day 3, cells were then subjected to the resazurin assay as above.

### Signal transduction assays

Serum was withdrawn from cultures of *Esyt2^+/+^* and *^−/−^* MEFs for 16 h prior to stimulation with bFGF (20 ng/ml)/heparin (5 μg/ml), EGF (100 ng/ml) or FBS (10%). Whole cell extracts were prepared using Triton lysis buffer (50 mM Tris [pH 7.5], 1% Triton X-100, 10% glycerol, 150 mM NaCl, 1 mM EDTA, 1 mM sodium orthovanadate, 1 mM phenylmethylsulfonyl fluoride, and 1 μg/ml of aprotinin, leupeptin and pepstatin), and cleared by centrifugation (20 min., 20,000 g, 4deg.C). Activation of ERK and AKT was examined by Western Blotting using 20 μg of protein extract and the antibodies to phospho-ERK1/2 (Sigma), phospho-AKT (Cell Signaling) and ERK2 (J. Grose). Immune complexes were detected using HRP-conjugated secondary antibodies and ECL+ (GE HealthCare).
